# Can remote infrared cameras be used to differentiate small, sympatric mammal species? A case study of the black-tailed dusky antechinus, *Antechinus arktos* and co-occurring small mammals in southeast Queensland, Australia

**DOI:** 10.1371/journal.pone.0181592

**Published:** 2017-08-09

**Authors:** Emma L. Gray, Todd E. Dennis, Andrew M. Baker

**Affiliations:** 1 School of Earth, Environmental and Biological Sciences, Science and Engineering Faculty, Queensland University of Technology, Brisbane, Qld, Australia; 2 School of Biological Sciences, Science Faculty, University of Auckland, Auckland, New Zealand; University of Sydney, AUSTRALIA

## Abstract

The black-tailed dusky antechinus (*Antechinus arktos*) is an endangered, small carnivorous marsupial endemic to Australia, which occurs at low population density along with abundant sympatric populations of other small mammals: *Antechinus stuartii*, *Rattus fuscipes* and *Melomys cervinipes*. Using *A*. *arktos* as a model species, we aimed to evaluate the effectiveness of infrared digital camera traps for detecting and differentiating small mammals and to comment on the broad applicability of this methodology. We also sought to understand how the detection probabilities of our target species varied over time and characterize their activity patterns. We installed 11 infrared cameras at one of only three known sites where *A*. *arktos* occurs for five consecutive deployments. Cameras were fixed to wooden stakes and oriented vertically, 35 cm above ground, directly facing bait containers. Using this method, we successfully recorded and identified individuals from all four species of small mammal known previously in the area from live trapping, including *A*. *arktos*. This validates the effectiveness of the infrared camera type and orientation for small mammal studies. Periods of activity for all species were highly coincident, showing a strong peak in activity during the same two-hour period immediately following sunset. *A*. *arktos*, *A*. *stuartii* and *M*. *cervinipes* also displayed a strong negative linear relationship between detection probability and days since deployment. This is an important finding for camera trapping generally, indicating that routine camera deployment lengths (of one-to-two weeks) between baiting events may be too long when targeting some small mammals.

## Introduction

Earth is currently experiencing biodiversity losses of a magnitude described by many as constituting a sixth mass extinction [1, 2]. Conservation of threatened cryptic species often hinges on our ability to identify where they occur, so that critical populations can be monitored and managed appropriately. Field surveys involving the capture of individuals by live traps is a common method employed by wildlife researchers to establish the occurrence of small terrestrial mammal species and to monitor populations (e.g., their abundance, survival and recruitment) over time [3, 4]. These direct sampling methods provide immediate and generally unambiguous species identifications and enable additional information to be collected from individuals, such as genetic material and their sex, age, body mass and condition [5]. However, recently, the proliferation of commercial wildlife camera traps has led to a sharp increase in the use of camera traps for small-medium sized mammal occurrence surveys and monitoring [6–8]. Camera traps are generally defined as remotely triggered cameras that automatically take images and / or videos of passing animals [6]. Remotely deployed cameras eliminate the need for researchers to trap and physically handle wild animals (thus avoiding often severe regulatory constraints) and can be deployed for long periods (up to several months), thereby reducing operational costs, time and effort [5, 7]. Although camera traps may be seen and / or heard by animals to some degree [9, 10], they still provide an opportunity to detect and monitor rare and / or trap-shy species that may otherwise be missed or under-detected by direct census methods [11, 12], as well as collect potentially valuable information about behaviour and activity [13, 14].

When deployed in the field, camera traps are generally mounted horizontally on trees, oriented passively outward or toward one or more bait holders [5, 15]. Such a setup has proved highly successful for detecting a wide variety of medium-large mammals, such as cats [16], ungulates [17] and foxes [18]. For detecting smaller mammal species, having the camera positioned closer to the subject is advantageous [19]. In such cases, whether the best camera orientation is horizontal or vertical is an open question (see [5, 15, 20, 21]). De Bondi et al. [5] demonstrated that small-medium sized mammals could be successfully detected by vertically orienting camera lenses toward a bait holder placed a standard height above ground. This alternative method has since been used to successfully detect the critically endangered central rock-rat (*Zyzomys pedunculatus*) [22] and invasive black rat (*Rattus rattus*) [12, 23]. However, the accurate identification of small mammals from camera traps is still challenging, especially where morphologically similar looking species co-exist [5, 24, 25]. If camera trapping is going to continue to be used for small threatened species surveys and monitoring, then further ‘proof of concept’ evidence is required in challenging environments to ensure that individual species can be consistently detected and identified where they occur, and that efforts to reduce field time and cost do not compromise estimates of species occurrence and persistence [24, 25].

The black-tailed dusky antechinus, *Antechinus arktos*, is one of 15 species of *Antechinus*, a genus of small (16–170 g) carnivorous marsupials endemic to Australia [26–28]. Antechinus are predominantly nocturnal insectivores renowned for their semelparous reproductive system, which features a short, promiscuous mating period, concluded by the abrupt death of all males in a population [26, 29]. Collectively, members of the genus occur in coastal / near coastal forest across all of Australia’s states and mainland territories. The geographic distributions of several species, including *A*. *arktos*, are severely limited [27]. The species is known only from three sites located a maximum 8 km (straight line distance) apart within cloud forest at the summit of the Tweed Shield Volcano caldera (900–1200 m elevation), which straddles the border of Queensland (Qld) and New South Wales (NSW) in mid-eastern Australia [30]. Therefore, *A*. *arktos* has been classified as Endangered in both states [31, 32] and is currently being considered for federal threatened species listing. Currently, the most important conservation priorities for *A*. *arktos* are to ensure the continued persistence of the three known populations and locate and protect previously unknown populations, should they exist. However, *Antechinus arktos* exemplifies the challenges associated with detecting and monitoring small, elusive / rare mammals.

Recently, a two-year mark-recapture study of *A*. *arktos* was undertaken using Elliott (metal box) traps [30]. This method proved effective at capturing *A*. *arktos*; however, trap success (number of captures divided by number of trap nights) was consistently low throughout its limited range, especially outside their short pre-breeding / breeding period, never exceeding 3.5% (or 7 individuals per 200 trap nights). Additionally, *A*. *arktos* co-occurs with much larger populations of the brown antechinus (*Antechinus stuartii)*, bush rat (*Rattus fuscipes*) and fawn-footed melomys (*Melomys cervinipes*), which may restrict access of *A*. *arktos* to live traps. Thus, to be 95% confident of detecting the species at other sites if they are present requires 600 trap nights conducted within this short enhanced detection-timing window [30, 33]. Deploying such a large number of traps at remote sites limits the number of sites that can be surveyed.

The camera trapping approach described by De Bondi et al. [5] may provide an alternative means of detecting *A*. *arktos* for occurrence surveys and monitoring programs. However, first camera trapping trials within known *A*. *arktos* habitat must be conducted. In contrast to many previous camera trapping studies, *A*. *arktos* co-occurs with a morphologically similar congener *A*. *stuartii* and other highly abundant small mammal populations, which may confound accurate identification. Therefore, using *A*. *arktos* as a model, we aimed to: 1) assess the utility of infrared digital camera traps for detecting and distinguishing *A*. *arktos* from other co-occurring small mammals; 2) identify factors that influence temporal variation in detection probability for each species; 3) examine diel activity patterns and their extent of overlap among species’ and 4) provide recommendations concerning the applicability of camera trapping as a survey method for *A*. *arktos* and other similar small mammal species.

## Methods

All aspects of the study were carried out with approval from the Queensland University of Technology ethics department (approval number: 1400000005) and the Queensland Parks and Wildlife service (approval number: WITK14454114).

### Study site

Our study was conducted at Best of All Lookout (28.2415°S, 153.2640°E) within Springbrook National Park, ~100 km south of Brisbane, Queensland, Australia. Located at the rim of the Tweed Shield Volcano caldera, Springbrook National Park has major remnants of UNESCO World Heritage listed ‘Gondwana Rainforests of Australia’ that provides important refugia for many relict and endemic species of flora and fauna [34]. Mean elevation of the site is 950 m and consists of complex notophyll vine forest (Regional Ecosystem 12.8.5) and simple microphyll fern forest (Regional Ecosystem 12.8.6) [35]. The study area was centred in a steep headwater gully containing the cloud-stripping stream lily, *Helmholtzia glaberrima*, and a small stand of Antarctic beech, *Lophozonia moorei*.

### Camera-trapping design

**Fig 1:**
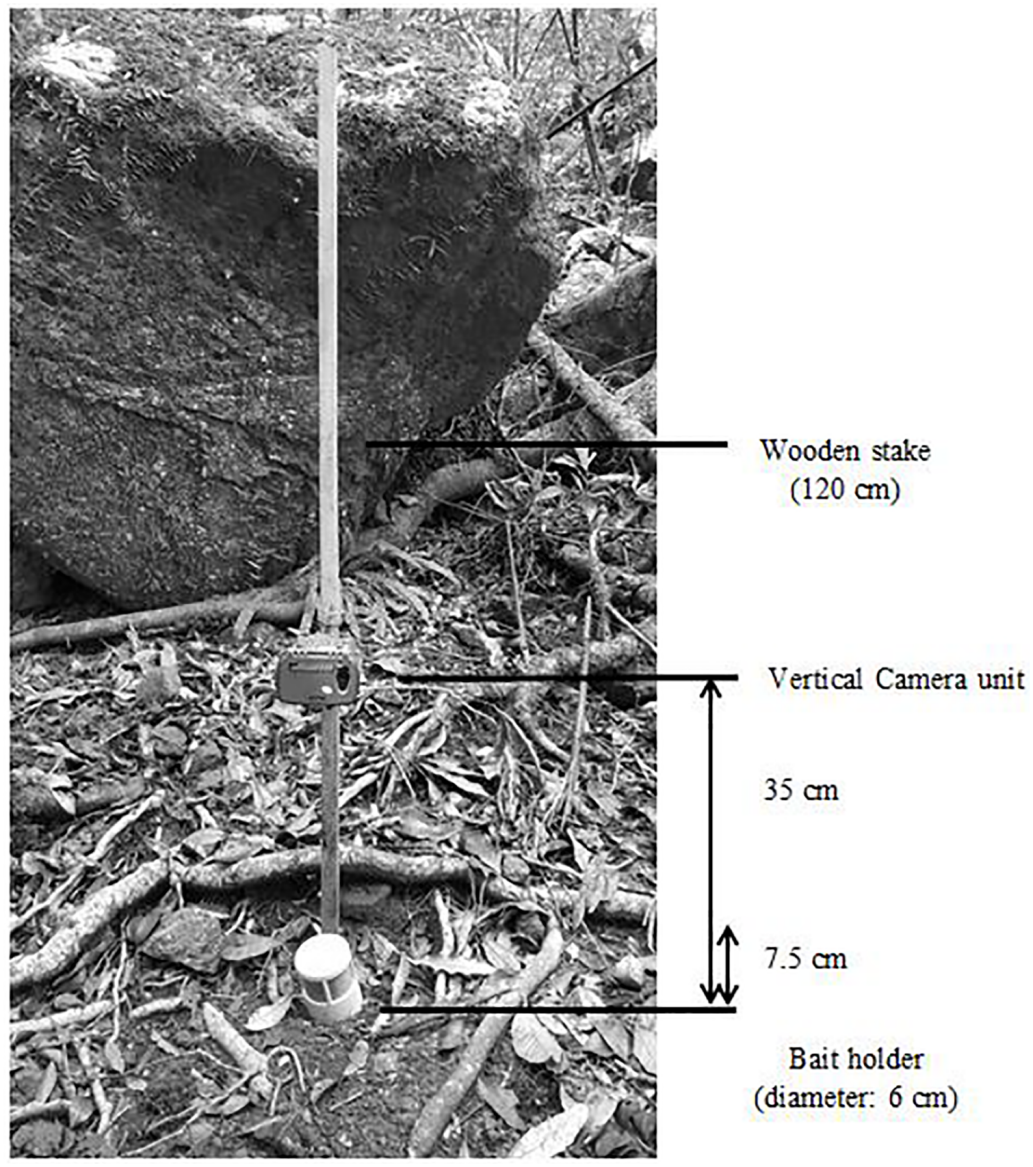
Schematic image of the vertical camera set-up showing camera positioning, with essential components and distances labelled.

Camera trapping was conducted between 10 August and 14 October 2016, which encompassed the breeding period from pre-mating to post-mating male die-off of the two local *Antechinus* spp. Eleven Ltl Acorn^®^ infrared digital cameras (Ltl-5310 series) were deployed at the study site and left in the same position for five consecutive deployments (ranging from 11–16 days in duration). Placement of the cameras utilized a pre-existing live-trapping grid consisting of 4 (200 m long and 20 m apart) parallel transects oriented down slope [30]. Cameras were randomly assigned to stations set ~50 m apart along each transect. We recognise this is a small area to deploy camera traps; however, *A*. *arktos* has a very limited known distribution and may occur patchily even within sites it is known to occur [30, 36]. Setting camera traps within the live-trapping grid, where the species has previously been captured, allowed us to be confident that *A*. *arktos* was present, and thus detectable, rather than absent due to unsuitable habitat. Cameras were mounted on wooden stakes that were hammered into the ground, with the lens positioned 35 cm above the ground surface and directed downward towards a bait container (Fig 1). To limit the occurrence of false positive camera detections, vegetation and leaf litter were cleared from within the camera’s infrared sensor zone. Several layers of cream-coloured masking tape were placed over the cameras’ LED lights to reduce the intensity of the flash when the cameras were triggered and avoid over-illuminating or possibly startling the target species. A seven-day field trial conducted in July 2016 at the study site was used to optimize camera settings and quantify the operational periods of the cameras. Based on information collected during this field trial, we configured all cameras to record a single photograph (JPEG format, 5 megapixels) when triggered, immediately followed by a 20-s video (AVI format, 640 by 480 pixels per frame), followed by a 10-min interval, 24 hrs per day, as some antechinus species are known to be at least partially diurnal [37, 38]. We considered a 10-min minimum interval to the next possible recording event of the cameras to be an acceptable compromise between detecting the same individuals repeatedly (which reduces battery life, as well as available storage of recorded media) and the likelihood of failing to record individuals that only infrequently visited the cameras’ monitoring areas [23]. To increase the probability of detecting target species (particularly *A*. *arktos*), we used a bait mixture of peanut butter, oats and bacon, which is commonly used in live trapping studies [3, 30] and has proven to be a superior bait type for antechinus [39], together with a single application (2-s spray) of FeralMone^™^, a generic carnivore attractant (Animal Control Technologies, Somerton, VIC, Australia; Jesse Rowland pers.comm.). Bait containers comprised 60 X 75 mm PVC vent cowls that were secured to the ground with tent pegs [40]. These devices permitted target species to see and smell the bait mixture but prevented them from directly accessing or removing it. Bait (including FeralMone), camera batteries and storage media (SD cards) were retrieved and replaced after each successive deployment.

Animal recordings were identified to species level based on body size, body and head shape, tail length, ear morphology and behaviour (particularly movements captured on video). Reference photographs of *A*. *arktos* and *A*. *stuartii* taken during live trapping aided in identification. If identification was still uncertain after viewing both image and video files the animal was classified as an “unknown mammal”, “unknown antechinus” or “unknown murid” for the purposes of categorisation and the record was not used in subsequent analyses. Because successful and consistent identification between the two *Antechinus* spp. was an important element of our study, all antechinus observations were independently assessed by two researchers (ELG and AMB) and only individuals positively identified by both researchers were categorised to species level.

Camera trap recordings were transcribed into separate binary response variables, one for each species per day of study, with a ‘1’ indicating species presence and a ‘0’ indicating species absence. Due to a high number of missing values (caused by the uneven deployment lengths ranging from 11–16 days) only the data from the first 11 days of each deployment were used for subsequent analyses, which was the minimum length of time that any of the cameras was set. We consider trimming deployment periods in the manner described above to be the best means of addressing missing observations, as plots of the capture proportions of each target species expressed as a function of the number of days of camera deployments clearly demonstrated that there were no marked changes in capture rates of any target species after day 11. Prior to statistical analysis we developed a list of explanatory variables that might reasonably influence detection probabilities (Table 1), and conducted exploratory data analysis on both the response and explanatory variables to test for outlier observations and evaluate the extent of collinearity among independent variables, as suggested by Zuur et al. [41].

**Table 1:** Explanatory variables used for modelling detection probabilities by remote camera traps of four species of small mammals at Springbrook National Park.

Variable Name	Term	Data type and values
**Cam**	Camera trap ID	Categorical: 11 levels (1,2,3,4,5 etc)
**Dep**	Deployment	Ordinal: 5 ordered levels (D1,D2,D3,D4,D5) each of 11 days length
**DayDep**	Days since deployment	Continuous: number of days since cameras were deployed
**Moon**	Moon phase	Categorical: 2 levels. M1 = light, first quarter to third quarter, M2 = dark, waning crescent to waxing crescent
**Rain**	Rainfall (mm)	Continuous: total rainfall (mm) measured per day from nearest weather station

### Data analyses

The data are repeated occurrence observations of the target species at fixed sites, with one level of spatial structure (Camera trap ID) and two levels of temporal structure (day of deployment nested within deployment). ‘Deployment’ can be considered to be both a study design and seasonal variable that reflects the breeding stage and survival of the two antechinus species over time. Camera deployments #1 and #2 occurred prior to antechinus breeding periods, deployment #3 coincided with breeding, and camera deployments #4 and #5 took place post-breeding. ‘Day of deployment’ reflects the duration of bait effectiveness and possible neophobic responses of species to the cameras within each deployment. Rainfall and moon phase were also included as possible explanatory variables due to their reported influence on the activity of other small mammal species [42].

We used generalized linear mixed models (GLMMs) to determine which variables best accounted for the detection probabilities of the target species. Because the response variable was binary, it was fitted to a binary distribution through a log-link function. Camera trap ID was included as a random effect nested within ‘deployment’ and ‘day of deployment’ to account for pseudoreplication (repeated measures) of observations over both time scales. Significance of fixed effects was assessed by computing Wald statistics [43]. The ‘best’ (minimal adequate) model was determined by fitting the full statistical model and then excluding non-significant terms using a stepwise backward selection process recommended by Crawley [43]. To examine the effects of individual explanatory variables on detection probability we plotted the fitted relationships from the minimal adequate model of each species. Following Rendall et al. [12] we also used estimated daily detection probabilities from the minimal adequate model for each species to calculate the cumulative detection probability for each day since deployment using the formula:

$$P=\;1\;–\;\left(1\;–p_{1}\right)\;*\;\left(1\;–p_{2}\right)\;*\;\left(1\;–p_{3}\right)\ldots \left(1\;–p_{n}\right)$$ (1)

Where *P* is the cumulative nightly detection probability, *p*_1_ is the detection probability for night one, and _n_ is the total number of survey nights per deployment. GLMMs were fitted using the R package lme4 [44].

### Activity patterns and overlap

Date and time records for each photo and video pair were used to investigate and compare the diel activity patterns of each target species. Because none of the target species had natural features or markings that allowed for individual identification within a species and because continued presence of species (after each 10-min interval) at a camera trap represented continued foraging activity, all capture records were included in the analyses as suggested by Carver et al. [45]. In addition, multiple records of individuals (e.g., two *R*. *fuscipes* individuals observed simultaneously at the same camera trap) were treated as separate events. All analyses of activity patterns were performed using the Overlap package [46] in R studio version 3.1.1 [47] using code adapted from Meredith and Ridout [46]. Probability density functions of activity for each species were estimated non-parametrically using kernel density estimates [13]. Then, to compare the activity times of each pair of sympatric species, the degree of overlap between the two estimated densities were measured. Various measures of overlap have been proposed; however, we used the ‘coefficient of overlapping (Δ)’ recommended by Ridout and Linkie [13]. This is a quantitative measure ranging from 0 (signifying no overlap) to 1 (signifying complete overlap). There are three alternative means of estimating the coefficient of overlapping, labelled Δ_1_, Δ_4_ and Δ_5_. Here, however, we used only Δ_1_ and Δ_4_, which were recommended for sample sizes less than 50 and sample sizes greater than 75, respectively [13, 46]. Confidence intervals for coefficients of overlapping were obtained as percentile intervals from a recommended 10 000 bootstrap samples [46].

## Results

### General findings

In total, 8 273 JPEG and AVI video pairs were recorded over 725 camera trap nights (Table 2). Of these pairs, 3 207 (38.8%) were deemed to be ‘false triggers’, with 49.9% caused by flies active during daylight hours. Additionally, 334 (4%) of observations could not be identified to species. This was overwhelmingly due to poor image quality, with less than six antechinus image and video pairs (across all cameras; 0.1%) unable to be identified to species level due to disagreement between researchers. The remaining 5 168 image and video pairs represented fauna from 10 different taxonomic groups, including our four target species: *A*. *arktos*, *A*. *stuartii*, *R*. *fuscipes* and *M*. *cervinipes*. Non-target animals included: northern brown bandicoot (*Isoodon macrourus*), possums (*Trichosurus spp*.), short-beaked echidna (*Tachyglossus aculeatus*), feral cat (*Felis catus*), macropod, and various rainforest birds (not identified). *R*. *fuscipes* was detected most frequently, constituting 56% of all mammal observations, with trap success (number of detections divided by number of camera trap nights) ranging from 77–98% between deployments. Next most numerous was *M*. *cervinipes* (15.2% of all observations; trap success ranging from 33–65%), *A*. *stuartii* (13.2% of all observations; trap success ranging from 15–59%), *I*. *macrourus* (3.9% of all observations; trap success ranging from 7–35%) and finally *A*. *arktos* (2.1% of all observations; trap success ranging from 3–21%). The two antechinus species could easily be distinguished from the Muridae species by their smaller body sizes and pronounced, pointed snouts (S1 Fig). Muridae could also be easily identified to species level due to marked differences in species facial features and ear morphology (*M*. *cervinipes* has a shorter face, while *R*. *fuscipes* has conspicuously rounded ears; S1 Fig). Distinguishing between the two antechinus species was more challenging. Generally, the much larger body size and more rounded rump of *A*. *arktos* was sufficient for definitive identification (S1 Fig). However, very large *A*. *stuartii* males are similar in body size to small *A*. *arktos* females [30]. In such cases, video footage was often crucial in order to confidently assign antechinus to species level. The behaviour and movements of the two antechinus species were particularly diagnostic. *A*. *stuartii* typically exhibited rapid stop-start movements and regularly climbed the wooden stakes supporting the cameras; in contrast, *A*. *arktos* tended to move in a much slower, ‘shuffling’ gait, and always remained on the ground.

**Table 2:** Summary of all camera trap image pairs recorded at Springbrook National Park over five successive deployments during 2016.

	Total No. captures	% of all mammal captures	Trap success %
***Rattus fuscipes***	2 439	56.0	77–98
***Melomys cervinipes***	662	15.2	33–65
***Antechinus stuartii***	574	13.2	15–59
***Antechinus arktos***	91	2.10	3–21
***Isoodon macrourus***	169	3.90	7–35
***Trichosurus sp*.**	81	1.90	2–22
***Tachyglossus aculeatus***	2	0.05	-
***Felis catus***	3	0.07	-
**Unidentified mammal**	134	3.10	-
**Unidentified murid**	134	3.10	-
**Unidentified antechinus**	66	1.50	-
**Macropod**	1	0.02	-
**Aves**	812	-	
**False trigger**	3 207	-	-
**Total**	8 375		

### Temporal changes in detection probabilities

**Fig 2:**
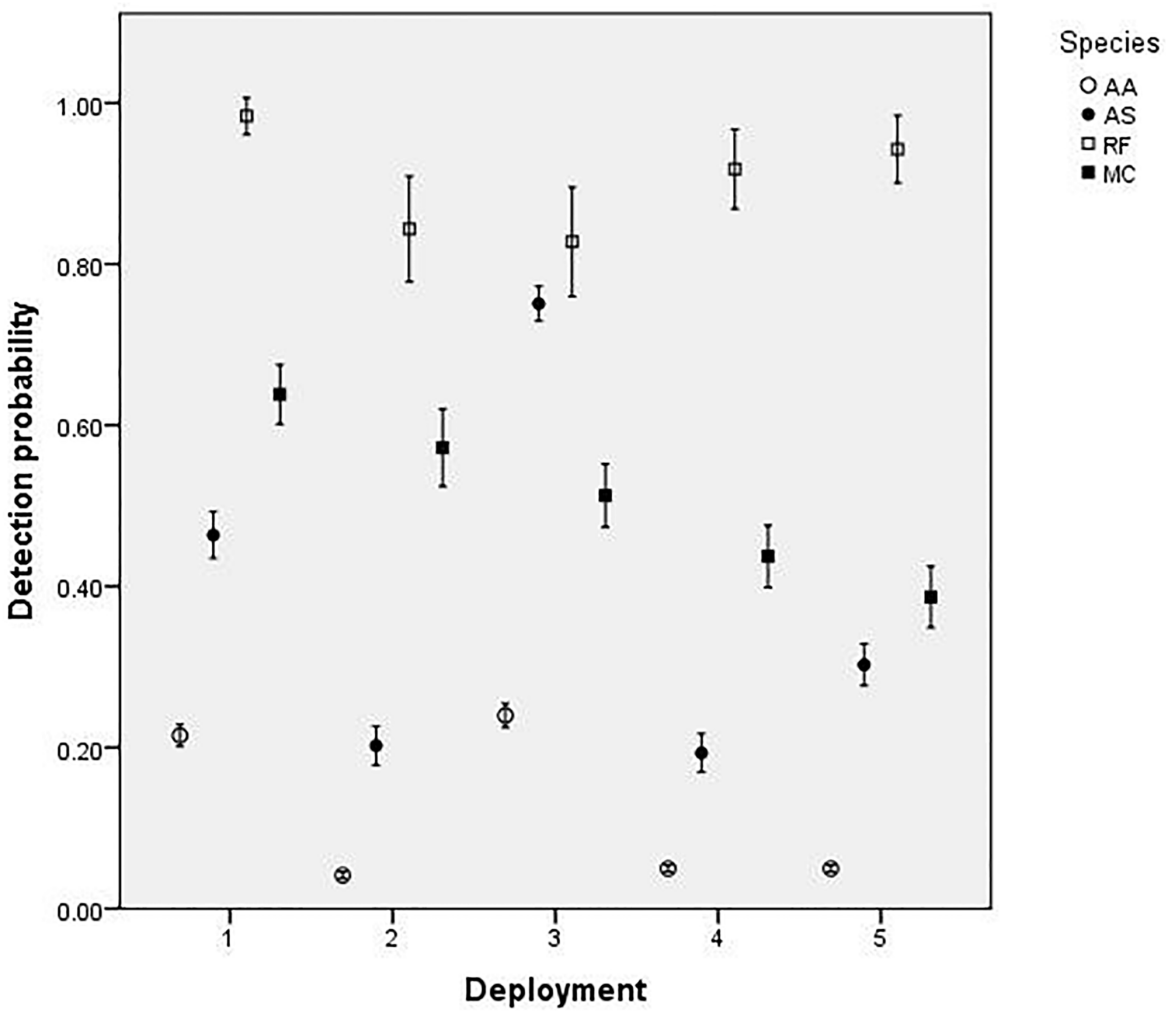
Model estimates of the effect of camera deployment period on detection probabilities of our study’s four small-mammal target species: *A*. *arktos* (AA), *A*. *stuartii* (AS), *M*. *cervinipes* (MC) and *R*. *fuscipes* (RF). Vertical bars represent 95% confidence intervals.

GLMMs of detection probability were fitted for each of the four target species. The minimal adequate detection model for *A*. *arktos*, *A*. *stuartii* and *M*. *cervinipes* included the fixed effects of ‘deployment’ and ‘days since deployment’ (Table 3). The most parsimonious detection model for *R*. *fuscipes*, however, was the null (Table 3), with detection probability consistently high (close to 1) irrespective of effects from any of the explanatory variables. *A*. *arktos* and *A*. *stuartii* both had significant quartic relationships with deployment number (Table 3); detection probabilities were lower during camera deployments #2, #4 and #5 compared with deployments #1 and #3 (Fig 2). In comparison, *M*. *cervinipes* had a strong negative linear relationship with deployment number (Table 3; Fig 2).

**Table 3:** Results of the minimal adequate model explaining detection of *A*. *arktos*, *A*. *stuartii*, *M*. *cervinipes and R*. *fuscipes*. Fixed factors include: Deployment (Dep) and days since deployment (DayDep). Deployment levels are labelled L, Q, C, and ^4, which stand for ‘linear’, ‘quadratic’, ‘cubic’, and ‘quartic’ polynomial terms respectively. Statistically significant values (*P* <0.05) are highlighted in bold font.

	Estimate	Std. Error	Z value	P value
*A*. arktos*				
**Intercept**	-1.481	0.274	-5.409	**<0.001**
**Dep.L**	-1.041	0.362	-2.880	**0.004**
**Dep.Q**	-0.054	0.326	-0.165	0.869
**Dep.C**	-0.593	0.422	-1.405	0.160
**Dep^4**	1.565	0.343	4.560	**<0.001**
**DayDep**	-0.147	0.044	-3.354	**<0.001**
*A*. *stuartii*†				
**Intercept**	0.404	0.264	1.531	0.126
**Dep.L**	-0.584	0.268	-2.181	**0.029**
**Dep.Q**	-0.443	0.268	-1.653	0.098
**Dep.C**	-0.076	0.288	-0.264	0.792
**Dep^4**	2.198	0.294	7.486	**<0.001**
**DayDep**	-0.166	0.032	-5.121	**<0.001**
*M*. *cervinipes*‡				
**Intercept**	1.935	0.236	8.210	**<0.001**
**Dep.L**	-1.115	0.261	-4.266	**<0.001**
**Dep.Q**	-0.054	0.255	-0.213	0.831
**Dep.C**	0.207	0.260	0.798	0.425
**Dep^4**	-0.103	0.256	-0.401	0.688
**DayDep**	-0.305	0.033	-9.167	**<0.001**
*R*. *fuscipes*§				
**Intercept**	13.669	1.439	9.496	**<0.001**

*A. *arktos* AIC: 401.4, BIC: 440.9, loglik: -191.7, deviance: 383.4, residual DF: 585

^†^*A*. *stuartii* AIC: 666.6, BIC 706.1, loglik: -324.3, deviance: 648.6, residual DF: 585

^‡^*M*. *cervinipes* AIC: 711.7, BIC: 751.2, loglik: -346.9, deviance: 693.7, residual DF: 585

^§^*R*. *fuscipes* AIC 157.8, BIC: 175.3, loglik-74.9, deviance: 149.8, residual DF: 590

**Fig 3:**
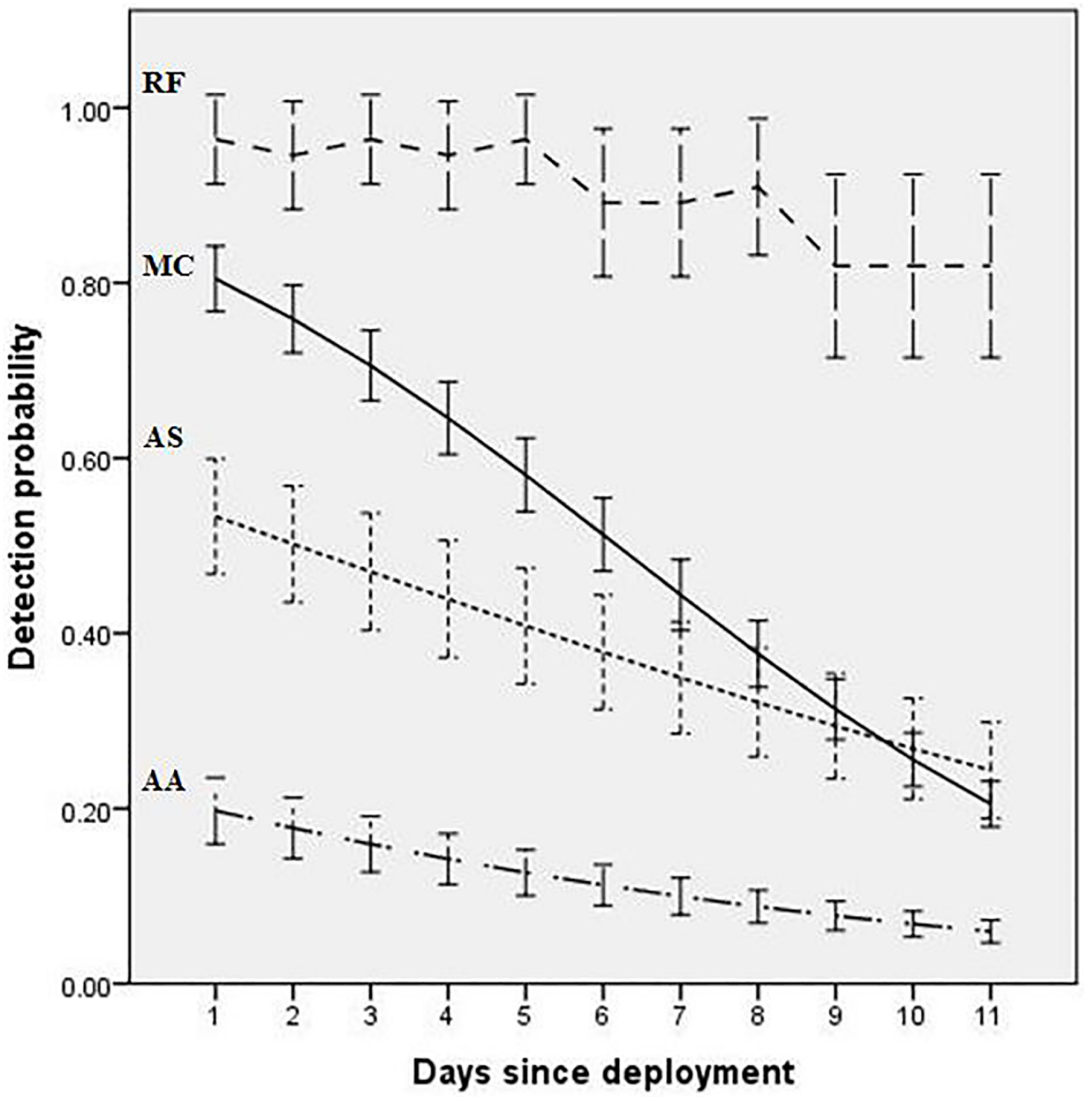
Relationships between detection probabilities estimated by GLMM and days since camera deployment (averaged across all five deployments) for our four target species: *A*. *arktos* (AA), *A*. *stuartii* (AS), *M*. *cervinipes* (MC) and *R*. *fuscipes* (RF). Bars represent 95% confidence intervals.

**Fig 4:**
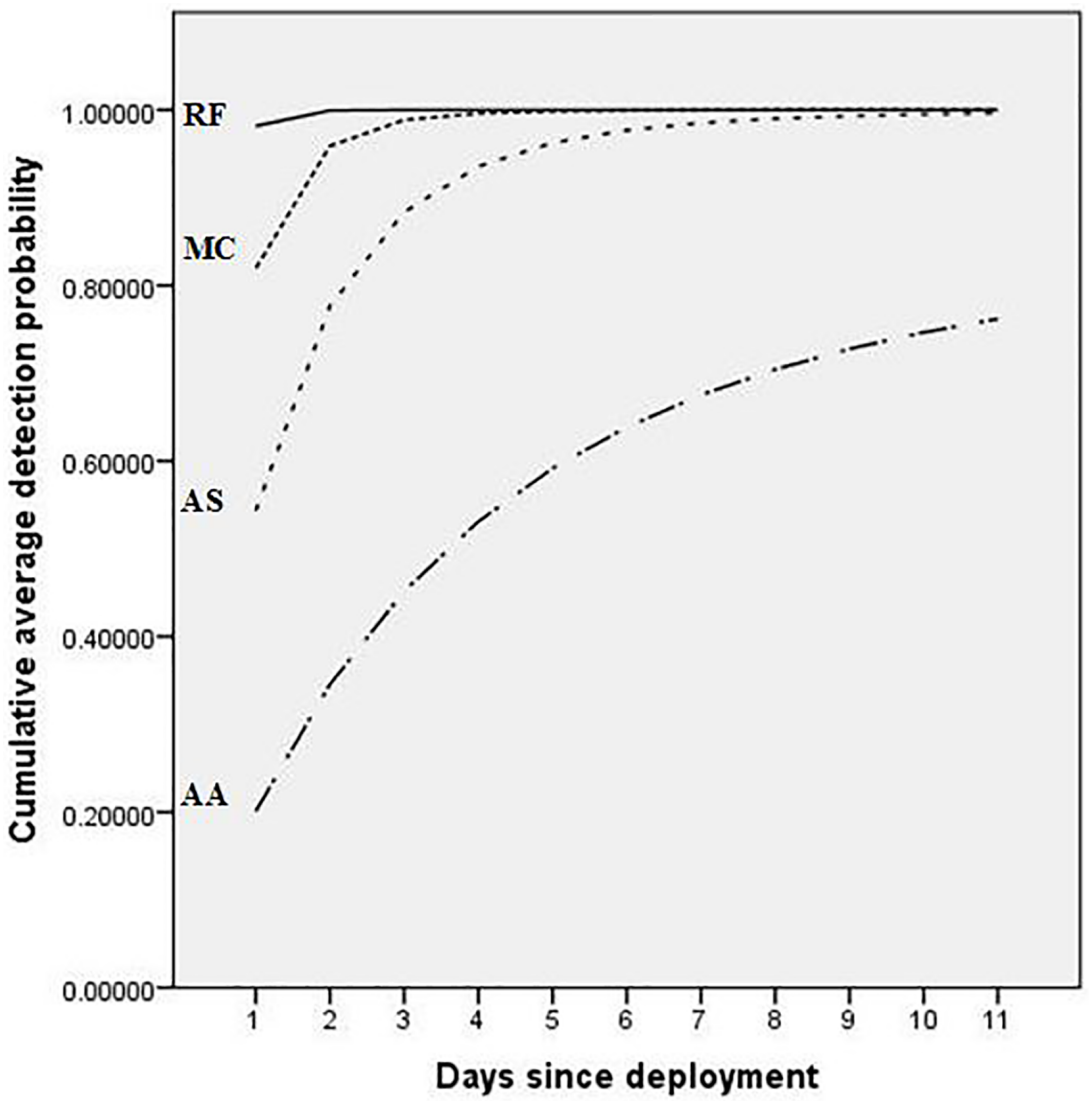
Cumulative detection probability curves calculated for *A*. *arktos* (AA), *A*. *stuartii* (AS), *M*. *cervinipes* (MC) and *R*. *fuscipes* (RF) from GLMM minimal adequate detection models (averaged across all five deployments).

Within individual camera deployments, there was a strong negative linear relationship between detection probabilities and ‘days since deployment’ for *A*. *arktos*, *A*. *stuartii* and *M*. *cervinipes*; the slopes of these relationships differed markedly between species (Table 3; Fig 3). *A*. *arktos* decreased from an average detection probability of 0.2 on the first day of each deployment to 0.06 on day 11, *A*. *stuartii* from 0.54 to 0.25 and *M*. *cervinipes* from 0.82 to 0.21 (Fig 3). Cumulative detection probability curves show that detection probability reached 95% after just one night for *R*. *fuscipes*, two nights for *M*. *cervinipes* and five nights for *A*. *stuartii* (Fig 4). However, a cumulative detection probability of 95% was never achieved for *A*. *arktos*. After 11 nights the cumulative detection probability of *A*. *arktos* was just 76% (Fig 4).

### Activity patterns and overlap

**Fig 5:**
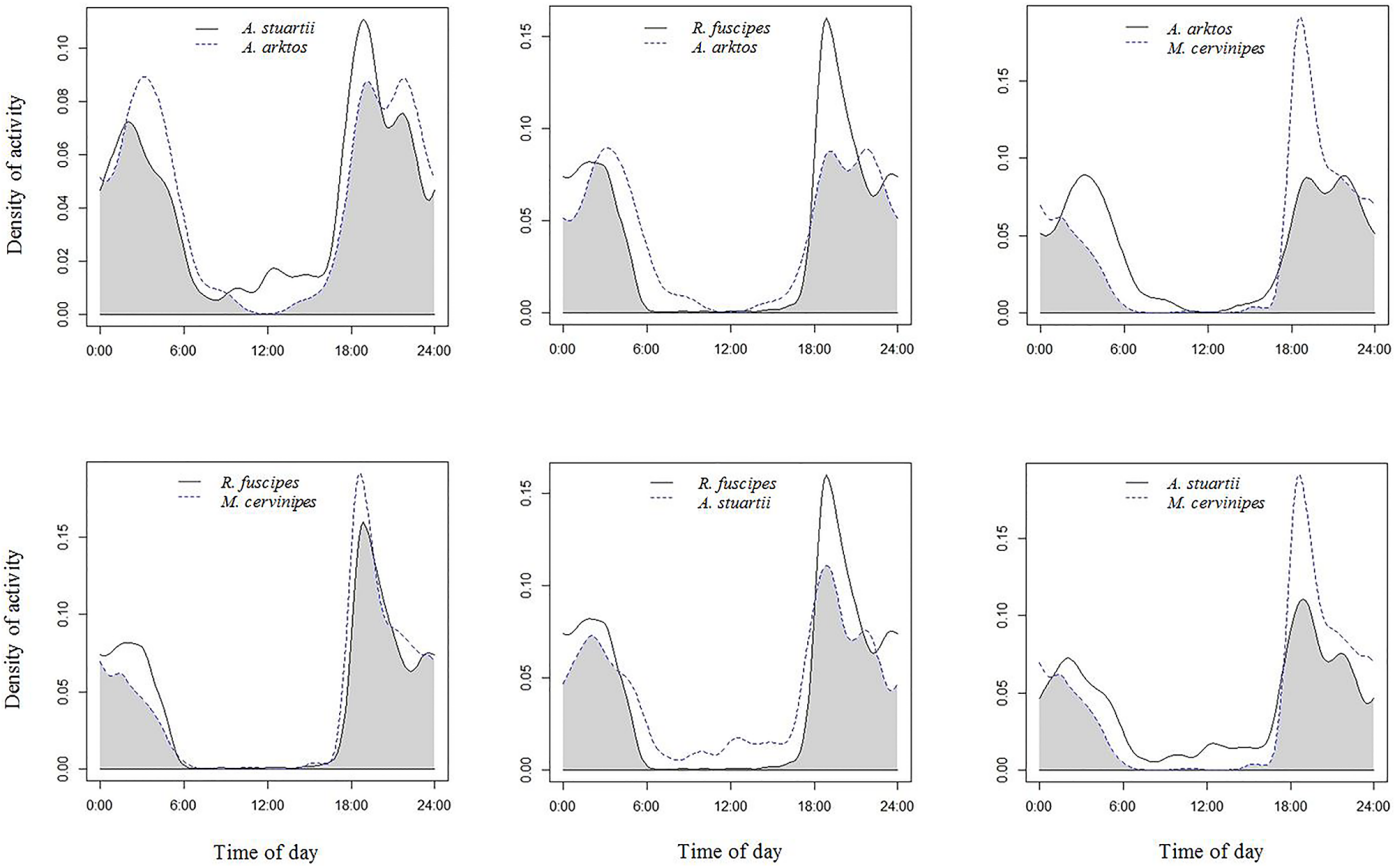
Estimates of the relative daily activity patterns for each pair of sympatric species pooled across all five deployments. On the x axis time is shown in 24 hour time. In each separate plot, the dashed and solid lines represent the kernel density estimates for the indicated species. The degree of activity overlap between the two species is the area under the minimum of the two density estimates, as indicated by the shaded area in each plot. The estimate of overlap and confidence intervals are given in Table 4.

Periods of activity were strongly coincident for all four target species, as indicated by values of overlap coefficients being >0.7 (Table 4). *A*. *arktos* was predominantly nocturnal (91% of observations were recorded during darkness), with a primary peak in activity between 18:00 and 19:00 hours, followed by smaller spikes of activity from 21:00 to 22:00 and at 03:00 hours, respectively (Fig 5). Similarly, *A*. *stuartii* had a primary peak of activity between 18:00 and 19:00 hours, with smaller spikes from 21:00 to 22:00 and at 02:00 hours (Fig 5). However, *A*. *stuartii* displayed stronger diurnal activity, with 115 detection events (20% of all captures) recorded during daylight hours. The majority of this daytime activity (86 captures) occurred during the species’ breeding period within deployment #3 from late August to mid-September. In comparison, *R*. *fuscipes* and *M*. *cervinipes* displayed a unimodal, peak in activity between 18:00 and 19:00 hours (Fig 5) and were strongly nocturnal, with 97% and 96% of captures recorded during darkness, respectively.

**Table 4:** Estimates of activity pattern overlap (0 = no overlap, 1 = complete overlap) between four co-occurring small mammal species, with sample size and approximate 95% bootstrap confidence intervals.

Species	Kernel Density	Sample size	CI 95%
***A*. *arktos*: *A*. *stuartii***	0.848	91	0.772–0.915
***A*. *arktos*: *R*. *fuscipes***	0.794	91	0.721–0.858
***A*. *arktos*: *M*. *cervinipes***	0.739	91	0.656–0.826
***A*. *stuartii*: *R*. *fuscipes***	0.804	570	0.771–0.837
***A*. *stuartii*: *M*. *cervinipes***	0.779	570	0.725–0.814
***R*. *fuscipes and M*. *cervinipes***	0.883	661	0.850–0.915

## Discussion

### Effectiveness of the camera trapping design

Our study confirms that remote infrared digital camera traps can be successfully used to detect and differentiate small, closely related, morphologically similar mammal species, including the endangered black-tailed dusky antechinus. Previous studies have highlighted the difficulties in distinguishing between small co-occurring mammalian species (including Muridae and antechinus) from images recorded by horizontally oriented camera traps [25, 39, 40, 48]. However, using a modified vertical mounting design established by De Bondi et al. [5] and extended here we conclude we could confidently identify most detections of small mammals to species. The vertical cameras captured dorsal perspectives of animals against a cleared ground surface, allowing critically diagnostic features such as body and head shape, tail length and shape, and ear morphology to be easily distinguished. The standardized dimensions of the bait holders and fixed distance of the cameras to the ground also provided a means to accurately estimate body size. Additionally, the 20-s video recordings corresponding to each still image allowed multiple angles of each individual to be viewed and its behaviour closely observed. We found that video recordings of behaviour (especially close-ups) were particularly useful and often allowed us to discriminate antechinus species with confidence. Although our findings are specific to the taxa and study area, the method could be applied to other small mammals such as civets, martens, shrews and other rodents.

However, despite the general success of this approach, we caution that prior live trapping at the site and / or a certain degree of familiarity with the target species was essential when attempting to taxonomically classify recorded individuals, even from paired monochrome images and video clips. We recommend that live trapping and field-based observations be used in concert with camera trapping, especially at new sites that have not been previously assessed. The present study also used two independent experts to examine all antechinus images and video, to improve accuracy of identification and ensure that the reviewer of the camera footage did not get complacent with identifications. Disagreement between independent experts resulted in the removal of just five possible antechinus detections from 665 paired images and video footage. The inclusion of a third independent reviewer may have allowed for a majority vote in these cases where the two experts could not agree. We advise that future studies employ as many independent reviewers as possible.

Our infrared cameras also recorded a large number of false triggers compared with other studies [5, 39]. This was likely due to the high infrared motion sensitivity of our particular camera model even at its lowest sensitivity. Most false triggers were of large flies moving near the bait stations during daylight hours. This issue can easily be avoided by configuring cameras to operate only during the night. However, in our case this would have meant foregoing collection of important data on the diurnal activity of *A*. *stuartii*. The wide detection zone of our particular camera model (Ltl Acorn^®^) may have also resulted in a number of false positive images, caused by animals triggering a camera trap outside of the cameras field of view [49]. In some instances, the trigger speed (time taken to take a photograph after it has detected heat/motion) of the cameras may also have been too slow for rapidly moving animals, resulting in out-of-focus or only partial images, which impeded classification to species. Use of ‘white-flash’ cameras may circumvent these problems. Unlike infrared illumination, white flash provides the ability to take colour images at night and thus enable unique pelage attributes of *A*. *arktos* (i.e., fuscous black hindfeet and tail, orange eye ring and cheek patch) to be used as an additional diagnostic feature in separating them from the more uniformly brown *A*. *stuartii*.

### Factors influencing detection probability

Changes to detection probability with time invariably differs from one species to another, depending on their level of attraction to baited camera traps and other key biological factors such as population density and activity [20]. Therefore, unsurprisingly *R*. *fuscipes*, which occurs at high population density at our and other sites where it is found [30, 50] and has a strong attraction to peanut butter and oat bait [39], had a near constant detection rate between and within camera deployments. In contrast, results of our GLMMs show that both deployment number and days since deployment are important factors influencing the detection probabilities of *A*. *arktos*, *A*. *stuartii* and *M*. *cervinipes*. Both *A*. *arktos* and *A*. *stuartii* exhibited marked differences in detection probabilities between camera deployments: probabilities were higher during camera deployments #1 and #3 (pre- and during breeding) than deployments #4 and #5 (post-breeding). This finding is consistent with previous live-trapping studies that found *Antechinus* spp. were significantly more trappable just prior to and during their breeding periods (owing to increased activity and movement during this time) and lowest post-breeding following the period of male die-off, when only pregnant females remain in the population [51, 52]. However, such low detection rates of both *Antechinus* spp. in our study during camera deployment #2 (when males were still alive), was unexpected. This period during the study experienced the highest total rainfall of any camera deployment (76 mm), which may have reduced the extent of activity in the antechinus [42, 53]. Moreover, periods of rainfall occurring early in the camera deployment may have degraded the olfactory attractiveness of our baits, similar to the reduced toxicity level of ‘1080’ bait after rainfall [54]. In contrast, we found that *M*. *cervinipes* exhibited a strong negative linear relationship of detection probability with respect to camera deployment number, suggesting either: 1) natural declines in either activity periods or population size occur between deployments; and / or 2) a persistent learned decrease of attraction to the bait, because the baits were inaccessible and thus offered no food reward, leading *M*. *cervinipes* to forage elsewhere for better opportunities. Optimal GLMMs for *A*. *arktos*, *A*. *stuartii* and *M*. *cervinipes* also identified a significant negative linear relationship between detection probability and days since camera deployment. Distinct peaks in detection rates on the first night of each camera deployment, when baits were fresh and most effective, suggest that the attractiveness of baits declines markedly over time [23].

Remote cameras have a distinct advantage over alternate survey methods such as live trapping because of the former’s longer operational periods without the need of human intervention [5, 55]. Ideally, with remote cameras such as those used in the present study, several weeks or even months of data can be collected with only two visits to a site: one to deploy the cameras and the second to retrieve or reset them. We noted that the initial high peak of interest in the camera trap baits (and lure combination) only lasted a single evening and was followed by progressively lower detection rates until the bait was replaced. To remain optimally effective, in our study baits would have needed to be refreshed every two-to-three days. For species with high initial detection rates over this time such as *R*. *fuscipes* (0.96–0.98), *M*. *cervinipes* (0.72–0.82) and *A*. *stuartii* (0.48–0.54), we found that a single camera deployment would be sufficient to achieve 95% cumulative detection probability if the species was present. Conversely, for rarer species such as *A*. *arktos* (detection rate 0.16–0.20), at least 14 survey days (five visits or successive three-day baiting deployments) would be necessary to achieve 95% cumulative detection probability if present. This extra effort would markedly add to the cost of an occurrence survey or long-term monitoring project for *A*. *arktos* and similarly rare species. This latter finding has general significance for other camera trapping studies and will be further discussed below.

### Diel activity and overlap

Information about the date and time that our cameras’ images and videos were recorded allowed us to examine the activity patterns of the four target species. Both *R*. *fuscipes* and *M*. *cervinipes* were found to be completely nocturnal, displaying a unimodal peak in periods of activity, consistent with previous studies of these species [37, 48, 50]. *A*. *arktos* and *A*. *stuartii* also exhibited primary peaks in activity, although smaller activity peaks were evident later in the night. Ours is the first study of activity patterns in *A*. *arktos*, a species which was discovered only very recently [27]. We found *A*. *arktos* to be primarily nocturnal, an activity pattern consistent with most of its congeners [26] but notably at odds with some other members of the Dusky antechinus species complex [28, 37, 38]. In contrast, *A*. *stuartii* showed greater diurnal activity during its breeding season (74% of all daytime observations occurred during this period). Such a shift in the timing of activity periods has also been reported for island populations of *A*. *minimus* and may be necessary to maximize reproductive success when competition among males for mating opportunities within populations is strong [38].

In general, we noted strong overlap of the activity periods in all four target species, with each displaying a peak in activity during the same two-hour period following sunset. In south-east Queensland, *R*. *fuscipes*, *M*. *cervinipes* and several *Antechinus* spp. are often sympatric and a certain level of interspecific competition is believed to occur [56]. The patterns of activity observed for the target species in our study suggest that diel temporal partitioning is not a mechanism used to promote coexistence. Rather, differences in microhabitat use and diet are likely to be the principal factors limiting competition among these species [36]. However, although we found no evidence of temporal partitioning in our study, a degree of avoidance at proximity may still occur. On several occasions (six and two times, respectively) video footage clearly showed *A*. *stuartii* fleeing from *R*. *fuscipes* and *A*. *arktos* to avoid confrontation. Dickman [57] suggested that evasive action by a subordinate before an encounter with a dominant species could be due to early detection, either by sound or smell. Such a behavioural strategy could occur between the larger *R*. *fuscipes* and *A*. *arktos* and the smaller *A*. *stuartii*.

### Applicability of camera traps as a survey method for rare or elusive species

Our study demonstrates that infrared digital camera traps can be used to detect and identify *A*. *arktos* and other small, morphologically similar mammals at a rate comparable to live-trapping. Although our study was not specifically designed to compare survey methods, three nights of live trapping were conducted in August 2016 (four days prior to the first camera trap deployment) and so some general comments are warranted. Live trapping (600 trap nights) captured 41 *A*. *stuartii*, 7 *A*. *arktos*, 77 *R*. *fuscipes* and 58 *M*. *cervinipes* (including recaptures). In comparison, the first three nights of camera trapping (deployment #1, 33 trap nights) recorded 68 *A*. *stuartii*, 15 *A*. *arktos*, 228 *R*. *fuscipes* and 117 *M*. *cervinipes*. Because live traps are limited to one capture per night, but cameras can record multiple individuals (and recaptures) per night, we expected that camera traps would record more species at more trap stations than live traps [58]. Future studies will aim to formally test the relative efficacy of live versus camera trapping for this species.

Nevertheless, such a high number of camera recordings from multiple stations highlights the potential use of this technique for other small mammal surveys and monitoring in the future. It is particularly relevant for areas inaccessible to large numbers of live traps or of high conservation value, where minimal disturbance to the target species is preferable. However, to achieve consistently high detectability of *A*. *arktos*, our work suggests baits need to be refreshed every two-to-three days, making the remote cameras more maintenance demanding than might be assumed. This limitation may not only apply to rare small mammals. Strongly declining rates of detection were also recorded in our study for *A*. *stuartii* and *M*. *cervinipes* and have been previously documented for other small mammals [22, 23, 39]. Examining fifteen camera trapping studies conducted on small-medium mammals over the last 10 years, we found that 80% deployed cameras longer than 1 week and 67% deployed cameras for at least 2 weeks before rebaiting / collection. This is an important finding for camera trapping generally, indicating that such routine camera deployment lengths may not be optimal when targeting some small mammals. For management of threatened mammals, the problem of declining bait effectiveness is more acute because on any given deployment, probability of detection is already low. It is uncertain if other attractants that have been used in camera trap studies, such as linseed oil, truffle oil or vanilla essence may enhance the time length of optimal attractiveness [39, 59, 60]. In any case, we recommend including the time since camera deployment as a covariate in future surveys of small mammals, especially when cameras are operational for extended periods. In cases of declining detection for rare fauna, a standard three night live trapping survey (where species can be identified at point of capture and other ecological information obtained) may be a more practical detection method than a camera trapping survey, which may require multiple, successive three-day baiting deployments.

## Supporting information

S1 Fig Example of the type of images obtained, and the ease of identifying diagnostic features (e.g., body size, body and head shape, and ear morphology) from vertically oriented cameras. Species include (clockwise from top left); A) *A*. *stuartii*, B) *A*. *arktos*, C) *R*. *fuscipes*, and D) *M*. *cervinipes*. Note antechinus are smaller in size and have more pronounced pointed snouts compared to the Muridae. *A*. *arktos* is larger, with a more rounded rump than *A*. *stuartii*; while, *M*. *cervinipes* has a shorter face than *R*. *fuscipes* and is smaller in size. *R*. *fuscipes* also has distinctive large rounded ears and coarser looking fur compared to *M*. *cervinipes*.
(TIF)

S1 Dataset Dataset used to generate GLMMs of detection probability for *A*. *arktos*, *A*. *stuartii*, *R*. *fuscipes* and *M*. *cervinipes*. (CSV)

S2 Dataset Dataset used to estimate the relative daily activity patterns of *A*. *arktos*, *A*. *stuartii*, *R*. *fuscipes* and *M*. *cervinipes* and measure their activity overlap. (CSV)
