# Supplementary figures and images for: Can remote infrared cameras be used to differentiate small, sympatric mammal species? A case study of the black-tailed dusky antechinus, Antechinus arktos and co-occurring small mammals in southeast Queensland, Australia
Source: PLoS One. 2017 Aug 9;12(8):e0181592. doi: 10.1371/journal.pone.0181592 (PMC5549885; doi:10.1371/journal.pone.0181592)

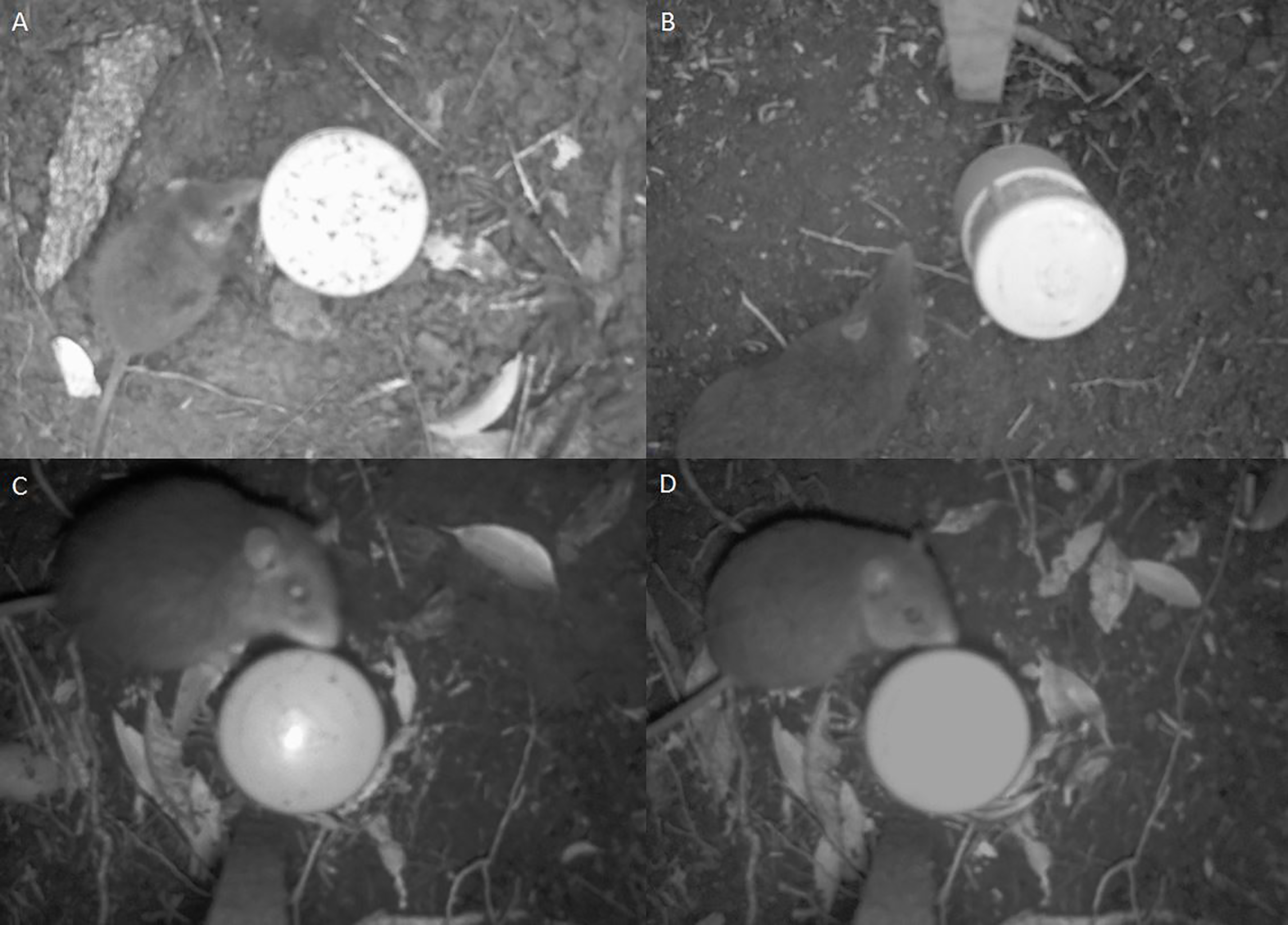

Supplement: S1 Fig — Species include (clockwise from top left); A) A. stuartii, B) A. arktos, C) R. fuscipes, and D) M. cervinipes. Note antechinus are smaller in size and have more pronounced pointed snouts compared to the Muridae. A. arktos is larger, with a more rounded rump than A. stuartii; while, M. cervinipes has a shorter face than R. fuscipes and is smaller in size. R. fuscipes also has distinctive large rounded ears and coarser looking fur compared to M. cervinipes. (TIF) [file pone.0181592.s001.tif]
